# Immortalized, premalignant epithelial cell populations contain long-lived, label-retaining cells that asymmetrically divide and retain their template DNA

**DOI:** 10.1186/bcr2754

**Published:** 2010-10-21

**Authors:** Karen M Bussard, Corinne A Boulanger, Frances S Kittrell, Fariba Behbod, Daniel Medina, Gilbert H Smith

**Affiliations:** 1 Mammary Biology and Tumorigenesis Laboratory, National Cancer Institute, National Institutes of Health, Building 37, Room 1112, 37 Convent Drive, Bethesda, MD 20892, USA; 2 Department of Cell Biology, Baylor College of Medicine, One Baylor Plaza, Houston, TX 77030, USA; 3 Department of Pathology and Laboratory Medicine, University of Kansas Medical Center, 3901 Rainbow Blvd, Lied G015, Kansas City, KS 66160, USA

## Abstract

**Introduction:**

During selective segregation of DNA, a cell asymmetrically divides and retains its template DNA. Asymmetric division yields daughter cells whose genome reflects that of the parents, simultaneously protecting the parental cell from genetic errors that may occur during DNA replication. We hypothesized that long-lived epithelial cells are present in immortal, premalignant cell populations, undergo asymmetric division, retain their template DNA strands, and cycle both during allometric growth and during pregnancy.

**Methods:**

The glands of 3-week-old immune-competent Balb/C female mice were used intact or cleared of host epithelium and implanted with ductal-limited, lobule-limited, or alveolar-ductal progenitor cells derived from COMMA-D1 pre-malignant epithelial cells. 5-Bromo-2-deoxyuridine (5-BrdU) was administered to identify those cells that retain their template DNA. Nulliparous mice were then either injected with [^3^H]-thymidine (^3^H-TdR) to distinguish 5-BrdU label-retaining cells that enter the cell cycle and euthanized, or mated, injected with ^3^H-TdR, and euthanized at various days after coitus. Sections were stained for estrogen receptor-α (ER-α) or progesterone receptor (PR) with immunohistochemistry. Cells labeled with both 5-BrdU and ^3^H-TdR were indicative of label-retaining epithelial cells (LRECs).

**Results:**

Cells that retained a 5-BrdU label and cells labeled with [^3^H]-thymidine were found in all mice and were typically detected along the branching epithelium of mature mouse mammary glands. Cells containing double-labeled nuclei (LRECs) were found in the intact mammary glands of both pregnant and nulliparous mice, and in mammary glands implanted with premalignant cells. Double-labeled cells (^3^H-TdR/5-BrdU) represent a small portion of cells in the mammary gland that cycle and retain their template DNA (5-BrdU). Some label-retaining cells were also ER-α or PR positive. LRECs distributed their second label (^3^H-TdR) to daughter cells, and this effect persisted during pregnancy. LRECs, and small focal hyperplasia, were found in all immortalized premalignant mammary-implant groups.

**Conclusions:**

The results indicate that a subpopulation of long-lived, label-retaining epithelial cells (LRECs) is present in immortal premalignant cell populations. These LRECs persist during pregnancy, retain their original DNA, and a small percentage express ER-α and PR. We speculate that LRECs in premalignant hyperplasia represent the long-lived (memory) cells that maintain these populations indefinitely.

## Introduction

In 1975, John Cairns proposed that, during the division of stem/progenitor cells, the template DNA strand of a parent cell is nonrandomly retained, whereas the newly synthesized strand is selectively segregated to a daughter cell [1]. As a result of this mechanism of asymmetric division, any spontaneous mutations or errors that may develop during DNA replication would occur in the newly synthesized strand and be passed along to the daughter cell, reducing the accumulation of genetic errors and, subsequently, cancer risk of the long-lived parent cell. In addition, this type of scheme would allow the survival and maintenance of progenitor "stem" cells that are capable of producing expendable daughter cells. Since then, many investigations have been carried out providing support for Cairns "immortal DNA strand" hypothesis [2-4] that include, among others, cells of the mammary gland and intestine [5,6].

The mammary gland is a unique organ that matures in the adult mammal by successive rounds of proliferation and apoptosis [7,8]. To accomplish this feat, a subpopulation of cells with regenerative properties is present in the gland. It was postulated that if mammary "stem" cells were present, these cells would retain an exogenously applied label, being then identified as the "longer-lived" cells of a population due to either mitotic quiescence or selective DNA segregation [9]. Although the state of differentiation was not clear (for example, pluripotent versus multipotent), it was evident that the mouse mammary epithelium contained cells that were the progenitors of the tissue [9]. It was found that some of these mammary progenitor cells were capable of retaining their label, and thus their template DNA strand, while they traversed the cell cycle [6]. Newly synthesized DNA was found to be distributed to daughter cells as a result of asymmetric cell division [6].

To identify progenitor cells as well as to determine whether asymmetric division occurs, two labels have been applied to cells over the course of mammary gland development [5,6,10]. In previous studies, 5-bromo-2-deoxyuridine (5-BrdU) was administered during allometric mammary gland growth and was used to identify those long-lived cells that were capable of retaining their label. A second DNA label, tritiated thymidine (^3^H-TdR), was used to distinguish those cells that were traversing the cell cycle at various periods during this study. Cells that contain a double-label of both 5-BrdU and ^3^H-TdR in their nucleus were interpreted as long-lived, label-retaining, cycling epithelial cells (LRECs).

Data have shown that various tissues within the body use asymmetric division for cellular replacement, protection of long-lived cells from mutations that could occur during DNA replication, and, consequently, from cancer risk [5,6,11-13]. However, it is unknown whether pre-cancer or cancer cells themselves use this method to maintain their populations. Earlier work in our laboratory demonstrated that retroviral insertions were maintained undisturbed through several transplant generations in nontransformed mammary populations despite the presence of actively replicating retrovirus and the presence of unintegrated retroviral DNA [14,15]. This scenario was found in mouse mammary tumor virus (MMTV)-induced epithelial hyperplastic outgrowths, which are immortal (that is, capable of unlimited growth during serial transplantation) [16]. Therefore, we hypothesized that long-lived epithelial cells present in immortal cell populations undergo asymmetric division, retain their template DNA strands, and cycle both during allometric growth and during pregnancy. We speculate that LRECs present in premalignant hyperplasia represent long-lived cells that maintain these preneoplastic populations indefinitely.

## Materials and methods

### Mice

Three-week-old, immune-competent female Balb/C mice were used as hosts for transplantation studies. All mice were housed in Association for Assessment and Accreditation of Laboratory Animal Care-accredited facilities in accordance with the NIH *Guide for the Care and Use of Laboratory Animals*. The National Cancer Institute Animal Care and Use Committee approved all experimental procedures.

### Cells

Clonal populations of immortalized, premalignant cells were derived by using flow cytometry for side-population (SP) cells from COMMA-D1 murine mammary epithelial cells (Kittrell *et al.*, unpublished data). COMMA-D1 murine mammary epithelial cells were originally isolated from the mammary glands of midpregnant Balb/C mice [17]. Derived clonal populations included "Non-Side Population 2" ("NSP2"), "Non-Side Population 3" ("NSP3"), and "Side Population 3" ("SP3"). The clonal population NSP2, interpreted to be a ductal progenitor cell, yields a ductal outgrowth that fills the mammary fat pad but does not differentiate in response to hormones (Kittrell *et al.*, unpublished data). NSP3 cells have poor growth *in vivo *and, when successfully implanted, typically fill less than 10% of the mammary fat pad with a nest of surviving cells (Kittrell *et al.*, unpublished data). Finally, the population SP3 cells typically fills 20% to 59% of the mammary fat pad on injection (Kittrell *et al.*, unpublished data). Cells were grown in Dulbecco's Minimal Essential Growth Medium (Gibco, Carlsbad, CA) mixed with F12 Nutrient Medium (Gibco), supplemented with 2% fetal bovine serum (Gibco), 1% 1 *M *HEPES Buffer (Sigma, St. Louis, MO), 1% 100× antibiotic antimycotic (Gibco), 5 ng/ml EGF (per 500 ml; Sigma), and 10 μg/ml insulin (per 500 ml; Sigma) at 37°C with 5% CO_2_.

### Cell transplantation

Smith *et al. *[18-20] previously described in detail the procedure used to clear the mammary epithelium from the inguinal fat pad of 3-week-old host mice, as well as the subsequent transplantation of cells. In brief, three mice per condition (six control (thoracic) glands; six inguinal fat pads) were anesthetized; both inguinal mammary fat pads, cleared; and a cell suspension, injected. Twenty thousand NSP2 cells, 50,000 NSP3 cells, or 50,000 SP3 cells suspended in phosphate-buffered saline were inoculated in 10-μl volumes by using a Hamilton (Reno, NV) syringe equipped with a 30-gauge needle. Control mammary glands (thoracic) remained intact.

### Cell labeling *in vivo*

**Figure 1:**
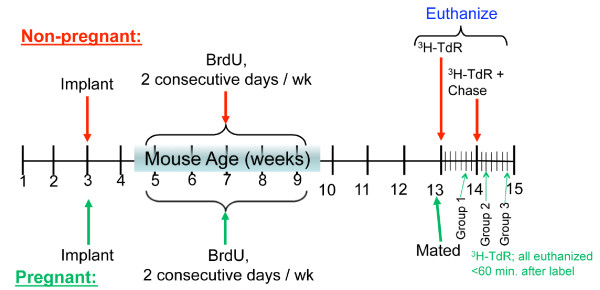
**Murine implantation experimental design**. The inguinal mammary glands of 3-week-old Balb/C mice were cleared of host epithelium and inoculated with either 20,000 (NSP2) or 50,000 (NSP3, SP3) immortalized, premalignant cells. Control (thoracic) number 3 and 8 glands remained intact. 5-BrdU was administered for 2 consecutive days/week for 5 weeks, followed by a break (chase) period of 3 weeks. Nulliparous female mice were then inoculated with ^3^H-TdR and subsequently euthanized within 90 minutes. The remaining mice were mated, inoculated with ^3^H-TdR, and euthanized within 90 minutes of receiving label at either 4 to 6, 8 to 10, or 12 to 15 days after coitus.

Two weeks after the implant surgery (mouse age of 5 weeks), 1 mg (100-μl volume), 5-bromo-2-deoxyuridine (5-BrdU) was administered via intraperitoneal injection for 2 consecutive days per week for 5 weeks (Figure 1). The 5-BrdU injections were then stopped for 3 weeks (mouse age, 13 weeks), when some nulliparous mice were inoculated with [^3^H]-thymidine (^3^H-TdR) (50 μCi, 100 μl) and euthanized within 90 minutes (Figure 1). Remaining mice (mouse age, 12 to 13 weeks) were mated with male Balb/C mice. Female mice were subsequently inoculated with ^3^H-TdR (50 μCi, 100 μl) and euthanized within 90 minutes at either 4 to 6, 8 to 10, or 12 to 15 days after coitus (Figure 1). Mammary fat pads that were either intact (that is, control thoracic) or containing the implanted cells were collected, fixed in neutral buffered formalin, and prepared for autoradiography and immunohistochemistry. Small intestine from the same mice served as a non-mammary control for autoradiography.

### Immunohistochemistry

Mammary glands, embedded in paraffin, were cut in 5-μm sections and mounted on positively charged slides. Sections were subsequently cleared in xylenes and rehydrated through a series of graded ethanols, and then heated to a boil in a microwave in 10 m*M *citrate buffer (BioGenex, San Ramon, CA) for antigen retrieval. Endogenous peroxidase activity was blocked by using a 0.6% hydrogen peroxide/methanol solution for 15 minutes at room temperature. Slides were blocked with normal goat serum (for estrogen receptor-α (ER-α) and progesterone receptor (PR) staining only) (Vector Laboratories, Burlingame, CA) for 20 minutes at room temperature, and then incubated overnight with either rabbit polyclonal anti-human progesterone receptor (1:1,600; Catalogue Number A0098; DAKO, Carpinteria, CA), rabbit polyclonal anti-human estrogen receptor-α (1:2,000; Catalogue Number sc-542; Santa Cruz, Santa Cruz, CA), or biotinylated anti-mouse BrdU (1:50; Catalogue Number A21301MP; Invitrogen). Negative tissue controls were included in all immunohistochemical analyses. For ER-α and PR staining, a secondary antibody of goat anti-rabbit (1:100; Vector Laboratories) was applied for 30 minutes at room temperature. Sections were then processed for 30 minutes at room temperature with the Vectastain ABC Standard Elite (Vector Laboratories), and visualized by using diaminobenzidine (DAB; Sigma, St. Louis, MO). Slides were counterstained with Gill's hematoxylin (Vector Laboratories).

### Autoradiography

After immunohistochemical processing, slides were transferred to distilled water and then coated with Kodak NTB-2 liquid emulsion (Carestream Health, Inc., Rochester, NY) diluted 1:1 with distilled water. After drying for 1 hour, slides were stored in lightproof slide boxes at 4°C for 7 days. After exposure, the slides were developed in Kodak D-19 (Carestream Health, Inc., Rochester, NY), washed in distilled water, and fixed in Kodak rapid fixer diluted 1:1 with distilled water. Slides were washed once more in distilled water and dehydrated through a series of graded ethanols and xylenes. Sections were mounted with Permount (Fisher Scientific, Pittsburgh, PA), and slides subsequently observed and evaluated for autoradiographic grains and for immunostaining.

### Cell labeling *in vitro*

Cells were plated in 25-mm^2 ^tissue flasks, cultured as described earlier, and allowed to incubate overnight. Cells were then treated with culture medium plus 0.5 μ*M *5-ethynyl-2-deoxyuridine (EdU) diluted in phosphate-buffered saline (PBS) for imaging by using the Click-iT EdU Imaging Kit (Invitrogen). Twenty-four hours later, the EdU solution was removed, cells were washed with PBS, and either the cells were immediately fixed at room temperature for 15 minutes with 4% paraformaldehyde, washed twice with Tris-buffered saline (TBS), and processed for EdU imaging, or the culture medium was replaced. Unfixed cells were then grown to confluence and serially passaged 5 times (NSP2 and NSP3 cells serially passaged at 1:3; SP3 cells serially passaged at 1:6). At the fifth passage, cells were washed with PBS and fixed with 4% paraformaldehyde at room temperature. Fifteen minutes later, the fixative was removed, and the cells were washed twice with TBS. For EdU imaging, cells were permeabilized for 20 minutes at room temperature with 0.2% Triton X-100 and subsequently washed twice with TBS. Freshly prepared Click-iT^® ^reaction cocktail (2 ml; Invitrogen, prepared as described by the manufacturer) per 25-mm^2 ^surface area was added to the cells, which were then incubated in the dark for 30 minutes. The reaction cocktail was subsequently removed, cells were washed twice with TBS, and nuclei were stained for 4 minutes at room temperature with diamno-2-phenylindole (1:1,000; DAPI; Invitrogen). DAPI was removed, cells washed twice with TBS, and flask bottoms were mounted by using Fluoromount-G (Southern Biotech, Birmingham, AL). EdU-positive cells were visualized by using fluorescence microscopy.

## Results

### Murine implantation experimental design

The mouse mammary gland is a dynamic organ that has been shown to regenerate itself from as little as one cell [14]. Consequently, it is likely that the mammary gland contains stem or progenitor cells allowing self-renewal to occur. Previously, it was shown that a population of cells exists within the epithelium of the mouse mammary gland that retains a ^3^H-TdR label, deemed label-retaining cells [9]. With this knowledge, we sought to identify and locate long-lived label-retaining mouse mammary epithelial cells *in vivo *as well as to determine the presence of long label retention in immortalized, premalignant cell populations maintained in epithelium-divested mammary fat pads. Three-week-old Balb/C female mice were anesthetized, and their inguinal mammary glands were cleared of host epithelium by using protocols described by Smith *et al. *[18-20] (three mice per condition; six cleared mammary fat pads). Either 20,000 or 50,000 immortalized, premalignant cells (NSP2, 20,000 cells; NSP3 and SP3, 50,000 cells) were inoculated in 10-μl volumes. Control (thoracic) glands remained intact (three mice per condition; six intact glands). Subsequently, to identify label-retaining cells present in the mouse mammary gland, 5-BrdU was administered to the 5-week-old female mice during allometric mammary ductal growth for 2 consecutive days per week for 5 weeks. The 5-BrdU injections were then stopped for 3 weeks (mouse age, 13 weeks). Some of the nulliparous female mice were then inoculated with ^3^H-TdR to distinguish 5-BrdU-label-retaining cells that remain in the cell cycle, and subsequently euthanized. The remaining 13-week-old mice were mated, inoculated with ^3^H-TdR, and euthanized at either 4 to 6, 8 to 10, or 12 to 15 days after coitus (Figure 1). Glands were collected and prepared for autoradiography and immunohistochemistry. Sections were further stained for ER-α or PR. Mammary cells within the ducts and alveoli that retain both a 5-BrdU and ^3^H-TdR double-label are indicative of long-lived label-retaining cycling epithelial cells (LRECs).

### Label-retaining cells were found in immortal cell populations

Sections of mouse mammary fat pads implanted with immortalized, premalignant cells were examined for evidence of outgrowths containing label-retaining cells. Of the implanted fat pads, 12 of 16 NSP2, 0 of 18 NSP3, and 10 of 18 SP3 yielded outgrowths that filled between 10% and 50% of the fat pad. In both pregnant and nulliparous mice, outgrowths containing NSP2-implanted cells typically filled 10% to 40% of the fat pad, yielding ductal structures with blunted side branches. Outgrowths containing SP3 cells filled 20% to 50% of the fat pad with both ductal and lobular structures. Finally, of specific note, NSP3 cells implanted into the mammary fat pads of athymic nude mice yielded only focal areas of epithelial cells. These results suggest that the NSP3 cells had indeed survived, but did not produce outgrowths consisting of ducts or alveoli or both, consistent with those found with NSP2 or SP3 cell implants. These spherical areas of epithelial cell growth typically filled only 3% to 5% of the fat pad (thus, it was not recorded as an outgrowth). In the NSP2 and SP3 outgrowths, occasional areas of atypical epithelial hyperplasia (~0.1 to 1.5 mm in diameter), with nuclei that appeared to be normal, were apparent (Table 1).

**Table 1:** Number of fat pads resulting in small focal hyperplasia of pregnant or nulliparous mice implanted with immortalized, premalignant cells

Cell type	Nulliparous	Pregnant
NSP2	6/6	6/10
NSP3	0/6	0/12
SP3	5/6	5/12

The number of fat pads containing small focal hyperplasia, versus those without, was visualized by using a light microscope and enumerated.

**Figure 2:**
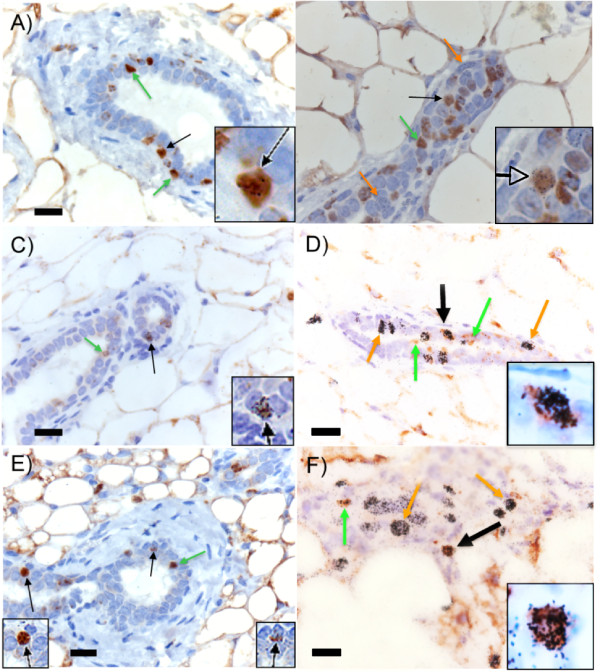
**Immortal cell populations contain LRECs**. Glands and fat pads from nulliparous mice either used intact or implanted with immortalized, premalignant cell clones were labeled with 5-BrdU (green arrow) and ^3^H-TdR (orange arrow). Double-labeled cells (labeled with both 5-BrdU and ^3^H-TdR, black arrow, inset) were identified by using light microscopy and were found in both intact glands **(a, c, e) **and implanted fat pads **(b, d, f)**. LRECs were found in all immortal, premalignant cells (b, NSP2; d, NSP3; f, SP3). Representative images are shown. Scale bars equal 20 μm.

Among the outgrowths and intact glands, cells labeled with 5-BrdU (long-lived label-retaining; template DNA strand) or ^3^H-TdR (cell cycling; newly synthesized DNA strand) were detected (Figure 2a-f: BrdU, green arrows; ^3^H-TdR, orange arrows). These single-labeled cells, which included 0.1% to 23.8% labeled with 5-BrdU and 0.4% to 14.3% labeled with ^3^H-TdR, were typically detected along the branching epithelium of mature mouse mammary glands in both pregnant and nulliparous mice (Table 2). Specifically, in the intact glands of mice during early pregnancy (4 to 6 days after coitus), we found that approximately 4.5% to 5.4% intralobular epithelial cells were labeled with ^3^H-TdR (cell cycling; newly synthesized DNA strand). At 8 to 10 days after coitus, the number of intralobular epithelial cells labeled with ^3^H-TdR decreased to 1.0% to 3.4% of the cells. By 12 to 15 days after coitus, alveolar differentiation was substantial, with 5.0% to 7.5% intralobular epithelial cells labeled with ^3^H-TdR. These results are consistent with those described by Traurig [21], who found that the highest rates of mammary epithelial cell proliferation occurred on day 4 after coitus and day 12 (after the onset of placental progestin secretion).

**Table 2:** Percentage of cells expressing either a 5-BrdU or ^3^H-TdR single label per field of view found in the glands and mammary fat pads of Balb/C female mice that were either intact or implanted with immortalized, premalignant cells

	Percentage (%)			
	
	Nulliparous		Pregnant	
		
Intact (control) gland:Implant group	5-BrdU	^**3**^**H-TdR**	5-BrdU	^**3**^**H-TdR**
Intact:NSP^2^	13.0:13.0	8.2:14.3	0.6:6.8	5.2:1.9
Intact:NSP^3^	23.8:19.7	3.5:2.0	0.8:3.5	2.7:8.7
Intact:SP^3^	12.0:4.4	4.8:0.4	0.1:4.0	1.9:6.5

The number of cells labeled with either 5-BrdU or ^3^H-TdR was counted in three random fields of view (400× magnification). In addition, the total number of cells in three random fields of view was determined (>1000 cells total counted; 400× magnification). The total number of cells labeled with either 5-BrdU or ^3^H-TdR was then divided by the total number of cells in a field of view, and the percentage of cells labeled with either 5-BrdU or ^3^H-TdR per field of view was obtained.

The frequency of epithelial cells labeled with ^3^H-TdR in fat pads implanted with NSP2 cells (ductal progenitor) was much less in pregnant mice than in nonpregnant (1.9% versus 14.3%, respectively; Table 2). These values were in contrast to fat pads implanted with the lobular progenitor cells NSP3 and SP3, in which the numbers of ^3^H-labeled cells increased in response to pregnancy. Cells containing double-labeled nuclei (both stained with 5-BrdU and containing autoradiographic grains (^3^H-TdR; LREC)) were found within all intact glands (Table 3) and were frequently located in mammary outgrowths containing implanted premalignant cells (Table 4). Representative images of double-labeled cells are shown in the insets of Figure 2a-f, which were similar for both nulliparous and pregnant mice whose glands were either intact or fat pads were implanted with immortalized premalignant cells.

**Table 3:** Percentage of double-labeled cells per field of view found in the intact glands of nulliparous or pregnant Balb/C female mice

	Percentage (%)	
	
Intact gland group	Nulliparous	Pregnant
Intact Group 1	0.02	0.04
Intact Group 2	0.3	0.004
Intact Group 3	0.1	0.02

The number of double-labeled (5-BrdU and ^3^H-TdR) cells was counted in three random fields of view (400× magnification). Next, the total number of cells in three random fields of view was determined (>1000 cells total counted; 400× magnification). The total number of double-labeled cells was divided by the total number of cells, and the percentage of double-labeled cells per field of view was obtained. The label "intact group" corresponds to the unoperated on (control) number 3 and 8 axillary mammary glands in each experimental group.

**Table 4:** Percentage of double-labeled cells per field of view found in the mammary fat pads of nulliparous or pregnant Balb/C female mice implanted with immortalized, premalignant cells

	Percentage (%)	
	
Cell-type group	Nulliparous	Pregnant
NSP2	0.15	0
NSP3	0.2	0.03
SP3	0	0.01

The number of double-labeled (5-BrdU and ^3^H-TdR) cells was counted in three random fields of view (400× magnification). Next, the total number of cells in three random fields of view was determined (>1000 cells total counted; 400× magnification). The total number of double-labeled cells was divided by the total number of cells, and the percentage of double-labeled cells per field of view was obtained.

### Label-retaining cells found in immortal cell populations asymmetrically divide

**Figure 3:**
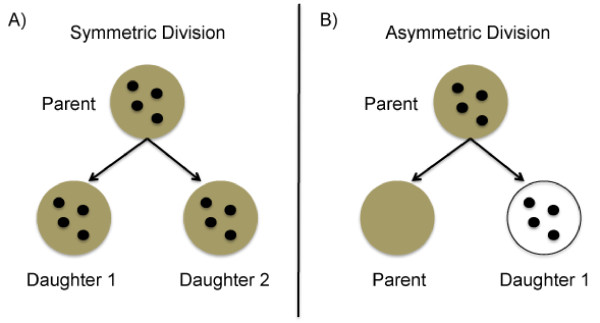
**Diagram of symmetric and asymmetric division**. Brown background indicates a cell with template DNA; black dots indicate a cell that is cycling. **(a) **During symmetric division, a cycling (black dots) parent cell with template DNA (brown background) divides equally, yielding two proliferating (black dots) identical daughter cells that each have one template DNA strand (brown background) and one newly synthesized DNA strand. **(b) **In asymmetric division, a cycling (black dots) parent (stem) cell with template DNA (brown background) undergoes unequal division, retaining its template DNA (brown background), but yielding one proliferating (black dots) daughter cell containing DNA that was newly synthesized from the parent's template strand.

**Figure 4:**
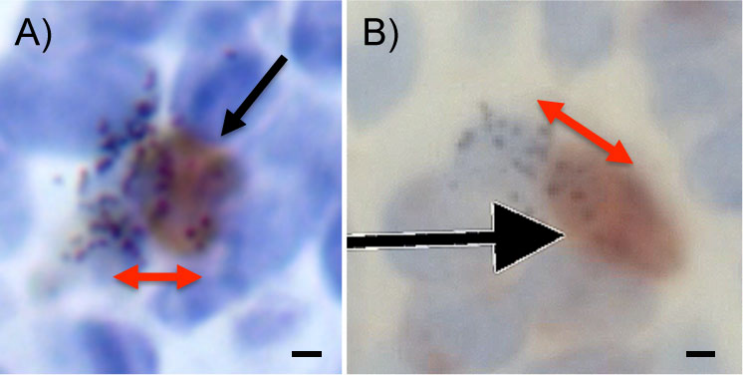
**Asymmetric cell division occurred in immortalized, premalignant cell populations**. Fat pads from mice implanted with immortalized, premalignant cells were labeled with 5-BrdU and ^3^H-TdR. Double-labeled cells that undergo asymmetric division and pass a ^3^H-TdR label on to their daughter cell (red arrow) in **(a) **nulliparous and **(b) **pregnant mice were identified by using light microscopy. Scale bars equal 5 μm.

Asymmetric division (Figure 3) would be particularly useful for stem and other long-lived cells, whereby a cell's archetype is retained for future generations. Being specifically interested in whether this phenomenon occurred within cells present in immortalized populations, we examined outgrowths from fat pads implanted with premalignant cells and labeled with both 5-BrdU and ^3^H-TdR. Asymmetric division with selective DNA segregation was rare, but occurred in the NSP2, NSP3, and SP3 cells that were implanted into cleared fat pads in both nulliparous (Figure 4a) and pregnant (Figure 4b) hosts. Furthermore, it was found that the number of immortalized, premalignant cells that contain a single label (either 5-BrdU or ^3^H-TdR) increased up to 108% after chase, providing further support that label-retaining cells within immortalized, premalignant populations undergo asymmetric division and pass the newly synthesized DNA strand to their daughters. A similar increase was observed in the intact control glands after the chase period.

### Epithelial cells in intact and immortal cell populations occasionally express ER-α or PR and take up ^3^H-TdR

**Figure 5:**
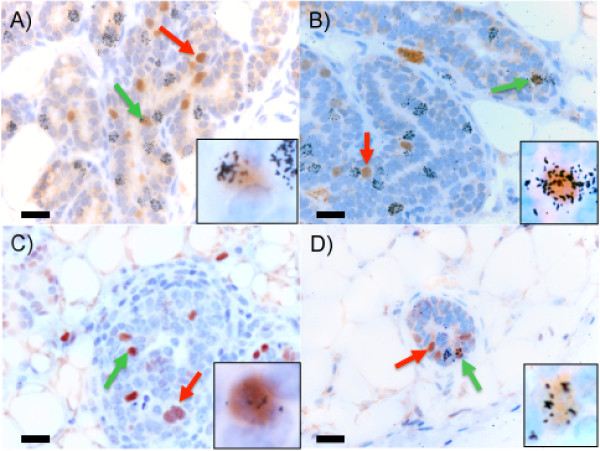
**LRECs present in the mammary fat pads of pregnant and nulliparous mice implanted with immortalized, premalignant cell populations express ER-α and PR and incorporate ^3^H-TdR into their nuclei**. Immunohistochemistry for ER-α (**(a, c)**, red arrow) and PR (**(b, d)**, red arrow), and labeling for ^3^H-TdR, was performed on mouse mammary glands (inguinal) of nulliparous mice (a, b) or pregnant (c, d) implanted with immortalized, premalignant cells. ^3^H-TdR was incorporated into the nucleus of some cells expressing the steroid receptors (green arrow, inset). Representative images are shown. Scale bars equal 20 μm.

It was next determined whether a percentage of epithelial cells present in immortalized, premalignant cell populations express estrogen receptor-α (ER-α) or progesterone receptor (PR). In the fat pads of nulliparous mice implanted with immortalized, premalignant cells, ER-α and PR expression was rare, being detected in 0 to 6% of immortalized cells counted per field of view (Figure 5a, c; Tables 5 and 6). In the intact glands of nulliparous mice, however, ER-α and PR expression was found in considerably larger cell numbers than in implanted fat pads: approximately 17% to 21% of cells counted per field of view (Supplemental figure S1 in Additional file 1; ER-α (a-c) and PR (d-f), red arrows) (Tables 5 and 6). These data demonstrate that although occasional cells express ER-α and PR, the number of immortalized, premalignant cells that express the steroid receptors is considerably smaller than the number found in normal mammary epithelium.

**Table 5:** Percentage of cells expressing ER-α per field of view found in the glands and mammary fat pads of Balb/C female mice that were either intact or implanted with immortalized, premalignant cells

	Percent (%)	
	
Intact (control) gland:Implant group	Nulliparous	Pregnant
Intact:NSP2	20.4:5.8	7.5:7.5
Intact:NSP3	21.5:6.1	7.4:5.9
Intact:SP3	16.4:2.3	6.8:6.4

The number of cells expressing ER-α was counted in three random fields of view (400× magnification). In addition, the total number of cells in three random fields of view was determined (>1000 cells total counted; 400× magnification). The total number of cells expressing ER-α was then divided by the total number of cells in a field of view, and the percentage of cells expressing ER-α per field of view was obtained.

**Table 6:** Percentage of cells expressing PR per field of view found in the glands and mammary fat pads of Balb/C female mice that were either intact or implanted with immortalized, premalignant cells

	Percentage (%)	
	
Intact (control) gland:Implant group	Nulliparous	Pregnant
Intact:NSP2	21.0:5.2	5.2:1.0
Intact:NSP3	18.2:0.6	5.8:0.0
Intact:SP3	17.8:0.0	6.8:0.8

The number of cells expressing PR was counted in three random fields of view (400× magnification). In addition, the total number of cells in three random fields of view was determined (>1000 cells total counted; 400× magnification). The total number of cells expressing PR was then divided by the number of cells in a field of view, and the percentage of cells expressing PR per field of view was obtained.

During pregnancy, ER-α was expressed in approximately 6% to 7.5% of the cells counted per field of view in fat pads with outgrowths of immortalized, premalignant cells (Figure 5b; Table 5). Conversely, PR expression in the immortalized, premalignant cells in pregnant mice was substantially less than ER-α expression: 0% to 1% of cells counted per field of view (Figure 5d; Table 6). In contrast, 5% to 7% of epithelial cells present within the intact glands of pregnant mice expressed ER-α and PR (Supplemental Figure 1) (Tables 5 and 6). Therefore, these data indicate that immortalized, premalignant cells implanted into the fat pads of pregnant mice that express PR are present in small numbers.

Cells positive for ER-α (Figure 5a and 5c; green arrows, inset) or PR (Figure 5b and 5d; green arrows, inset) and a ^3^H-TdR label were rare (<1%) both in outgrowths of immortalized, premalignant cells and in the intact gland (Tables 7 and 8). Nevertheless, these results suggest that some of the cells expressing the hormone receptors were also progressing through the cell cycle.

**Table 7:** Percentage of cells expressing an ER-α and ^3^H-TdR double-label per field of view found in the glands and mammary fat pads of Balb/C female mice that were either intact or implanted with immortalized, premalignant cells

	Percentage (%)	
	
Intact (control) gland:Implant group	Nulliparous	Pregnant
Intact:NSP2	0.20:0.10	0.13:0.01
Intact:NSP3	0.04:0.05	0.08:0.03
Intact:SP3	0:0.09	0.06:0.06

The number of cells expressing an ER-α and ^3^H-TdR double-label was counted in three random fields of view (400× magnification). In addition, the total number of cells in three random fields of view was determined (>1000 cells total counted; 400× magnification). The total number of cells expressing an ER-α and ^3^H-TdR double-label were then divided by the total number of cells in a field of view, and the percentage of cells expressing an ER-α and ^3^H-TdR double-label per field of view was obtained.

**Table 8:** Percentage of cells expressing a PR and ^3^H-TdR double-label per field of view found in the glands and mammary fat pads of Balb/C female mice that were either intact or implanted with immortalized, premalignant cells

	Percentage (%)	
	
Intact (control) gland:Implant group	Nulliparous	Pregnant
Intact:NSP2	0.20:0.02	0.04:0.006
Intact:NSP3	0.0:0.05	0.17:0.0
Intact:SP3	0.10:0.0	0.07:0.02

The number of cells expressing a PR and ^3^H-TdR (double-label) were counted in three random fields of view (400× magnification). In addition, the total number of cells in three random fields of view was determined (>1000 cells total counted; 400× magnification). The total number of cells expressing a PR and ^3^H-TdR double-label was then divided by the total number of cells in a field of view, and the percentage of cells expressing a PR and ^3^H-TdR double-label per field of view was obtained.

### Label-retaining cells were present in nonmammary tissues

Although it is evident that label-retaining epithelial cells are present in the regenerating mammary gland, it may be possible that these cell populations are also present in other tissues. Booth *et al. *[10] described that label-retaining cells were found in several nonepithelial mammary tissues, including nerve, fatty stroma, and endothelial tissue. Consistent with that observation, label-retaining cells, in this study, were also located among cartilage (Supplemental figure S2a in Additional file 2), adipose tissue (Supplemental figure S2b in Additional file 2), skeletal muscle (Supplemental figure S2c in Additional file 2), and periductal cells (Supplemental figure S2d in Additional file 1). These results indicate that label-retaining cells are present in tissues in addition to the mammary epithelium.

### Label-retaining cells were present in the premalignant cell populations *in vitro*

**Figure 6:**
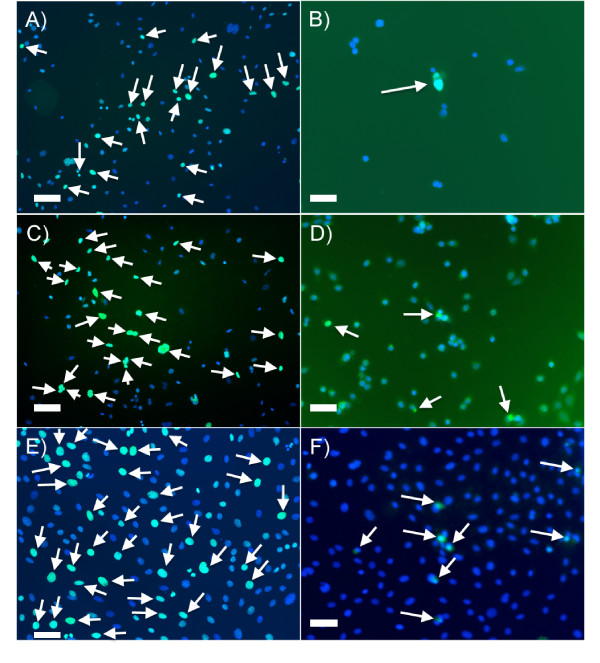
**Label-retaining cells were present in immortalized, premalignant cells *in vitro***. Immortalized, premalignant cell populations NSP2 **(a, b)**, NSP3 **(c, d)**, and SP3 **(e, f) **were cultured *in vitro *as described in Materials and methods. Cells were plated, allowed to incubate overnight, treated with 0.5 μ*M *EdU for 24 hours, and fixed, permeabilized, labeled with Alexa Fluor^® ^488, and counterstained with DAPI either (a, c, e) immediately or (b, d, f) after serial passage. Representative images are shown. Scale bars equal 40 μm.

To verify whether label-retaining cells were present in immortalized, premalignant cell populations *in vitro*, clones were cultured and treated with 0.5 μ*M *EdU. EdU-labeled cell nuclei were found in all immortalized, premalignant cell populations *in vitro *at both passage 0 and passage 5. In passage 5 cell cultures, a twofold to 5.5-fold decrease was found in the number of EdU-label retaining cells present compared with passage 0 (Table 9; Figure 6). One cause for the decrease in number of label-retaining cells might be the result of dilution of noncycling cells during serial passage. At passage 0, approximately 20% of immortalized, premalignant cells are EdU positive. If dilution of noncycling cells were in play, then, after five passages, cells seeded at a 1:3 (NSP2, NSP3) or 1:6 dilution (SP3) would have one cell in 243 (3^5^) or one cell in 7,776 (6^5^), respectively, as a label-retaining cell. This was not the case, as five NSP2 cells of 34 cells (14.7%), seven NSP3 cells of 157 cells (4%), and 21 SP3 cells of 561 cells (3.8%) were positive for EdU after five serial passages (Table 9). These results show that the label-retaining, premalignant populations are selectively preserved throughout serial passage *in vitro*. These data imply that label-retaining, premalignant cells were not out of the cell cycle during passage, but nevertheless retained their original DNA label. In addition, label-retaining cells in the fifth passage were occasionally found juxtaposed to unlabeled cells suggesting selective label retention during mitosis (Figure 6).

**Table 9:** Percentage of label-retaining cells *in vitro*

	Percentage of cells expressing EdU	
	
Cell type	Passage 0	Passage 5
NSP2	17.4	14.7
NSP3	23.2	4.0
SP3	21.0	3.8

The number of cells expressing EdU was counted in three random fields of view (100× magnification). In addition, the total number of cells in three random fields of view was determined (>100 cells total counted; 100× magnification). The total number of cells expressing EdU was then divided by the total number of cells in a field of view, and the percentage of cells expressing EdU per field of view was obtained.

## Discussion

Our data demonstrate that a subpopulation (<1%) of long-lived cells (LRECs), both in immortalized, premalignant cells and in the normal mouse mammary gland, maintain and protect template DNA through selective strand segregation during cell division. During pregnancy, the LRECs are stimulated to enter the cell cycle and contribute to new progeny through asymmetric divisions. We postulate that these cells represent long-lived cells (LRECs) are the source of regenerative capacity for both normal and premalignant epithelial populations. In a sense, this subpopulation of long-lived cells represents the "memory" or "repository" cells of these populations.

Here, we show that both untransformed mammary epithelial cells and immortalized, premalignant epithelial cells divide asymmetrically (Figure 4) and are selectively conserved through serial passage *in vitro *(Figure 6), suggesting that a subpopulation of progenitor-like cells resides in each of these populations. Is it the case that these label-retaining epithelial cells may be pluripotent or multipotent stem cells, giving rise to progeny committed to a specific lineage? Although the answer to that question is presently unknown, in this study, it was shown that LRECs are capable of self-renewal, a characteristic specific to stem cells, as identified by retention of a 5-BrdU label. Cells in the small intestine, neural tissue, skeletal muscle, and *Drosophila *ovarioles, as well as the mammary gland, have been shown to retain their template DNA selectively during asymmetric divisions [6,10,22]. In these cases, all of the chromatids possessing template DNA are retained. What is the mechanism(s) by which this is accomplished? Several reports have indicated possible mechanisms by which older DNA chromatids may be recognized and selectively retained. In fission, yeast kinetochore-specfic proteins associate selectively with the older chromatids during meiotic divisions [23]. In mouse colon crypt epithelial cells, sister chromatids were nonrandomly segregated during mitotic divisions, apparently by recognition of specific DNA sequences [24]. In addition, Armakolas *et al. *[25] suggest that individual old and new DNA strands may be selectively distributed during mitotic division in differentiating cells of different lineages. For example, selective strand segregation was found in endodermal cells, whereas random strand segregation was noted in others [25]. Thus, it may be possible that the cell type or tissue microenvironment or both regulate the mode of strand distribution, and therefore the pattern of cellular differentiation.

In addition to the mammary epithelium, label-retaining cells were also found in nonepithelial mammary structures. Booth *et al. *[10] discovered label-retaining cells in nerve, fatty stroma, and endothelial tissue. Here, label-retaining cells were also found within cartilage, adipose tissue, skeletal muscle, and in periductal cells (Supplemental Figure S2a-d in Additional file 2). These results suggest that progenitor cells may be present elsewhere within the body, serving as tissue reserve cells and being used only when needed. An example of such a cell can be found in skeletal muscle [13,26]. Shinin *et al. *[13] identified a subpopulation of muscle satellite cells that divided asymmetrically and retained their template DNA during muscle regeneration. It was found that these effects persisted during muscle growth, as well as during muscle injury [13]. At the present, though, it is unknown whether a specific physiological event, such as wounding, pregnancy, or cell death, is necessary to trigger the activation of tissue-specific progenitor cells. Nevertheless, their presence in the tissues identified in this study is intriguing.

Cells expressing either ER-α or PR also incorporated ^3^H-TdR into their nuclei (Tables 7 and 8), indicating that some steroid receptor-positive cells are traversing the cell cycle. These results are consistent with those found by Booth *et al. *[10], who surmised that the epithelial cells that express either ER-α or PR and incorporate ^3^H-TdR into their nuclei represent a functionally distinct cell population undergoing asymmetric division [10]. Thus, these results suggest that within the mammary gland, distinct cell populations exist (for example, ER-α and/or PR positive versus ER-α and/or PR negative), each potentially arising from a different progenitor.

Data presented in this study indicate that only a small number (<1%) of cells present in hyperplasia possess these progenitor-like cell characteristics. We believe it is possible that the lower frequency of double-labeled cells recorded in the present experiments may be due to the relatively short pulse used to introduce the second DNA label into the tissue, especially if cycling LRECs pass more slowly through the S phase than do symmetrically dividing cells. In past experiments [6], the second label was applied over a 48-hour period (in the presence of estradiol, estradiol plus progesterone, or estradiol, progesterone, plus prolactin), yielding a population of nearly 2% double-labeled cells [6]. Labeling for more than 48 hours may likely result in labeling both slowly cycling and rapidly cycling cells. Alternatively, it may be the case that immortalized, premalignant populations already contain progenitor cells whose template DNA strand was not initially labeled by 5-BrdU. In this case, the unlabeled template strand would be selectively retained, and the newly synthesized strand, labeled with 5-BrdU, would be passed to a daughter cell through asymmetric division. Thus, the number of LRECs (tagged by 5-BrdU) would be small. In the data presented here, however, we suppose that symmetric expansion of the progenitor population occurs, rendering this supposition unlikely.

## Conclusions

These findings demonstrate that a subpopulation of long-lived cells, characterized by their ability to retain a DNA label, are present in immortalized, premalignant cells. These cells retain their original DNA strands and divide asymmetrically to maintain their cell populations and protect their template DNA. During pregnancy, where these cells persist, they are stimulated to enter the cell cycle and contribute to new progeny through asymmetric divisions. New long-lived label-retaining cells, which include those that express ER-α and PR, continue to cycle. We speculate that LRECs present in premalignant hyperplasia represent the long-lived (memory) cells that maintain these populations indefinitely.

## Abbreviations

5-BrdU: 5-bromo-2-deoxyuridine; DAPI: diamno-2-phenylindole; EdU: 5-ethynyl-2-deoxyuridine; ER-α: estrogen receptor-alpha; ^3^H-TdR: [^3^H]-thymidine; LRC: label-retaining cell; LREC: label-retaining epithelial cell; MMTV: mouse mammary tumor virus; Non-SP2: non-side population 2; Non-SP3: non-side population 3; PBS: phosphate-buffered saline; PR: progesterone receptor; SP3: side population 3.

## Competing interests

The authors declare that they have no competing interests.

## Authors' contributions

GHS and CAB conceived the study, the design, and interpreted the data. KMB performed data collection, data interpretation, and wrote the manuscript. CAB and GHS performed the mouse surgeries. DM, FB, and FSK isolated and characterized the immortalized, premalignant COMMA-D cell clones. All authors have read and approved the final manuscript.

## Supplementary Material

Additional file 1 **Supplemental figure S1**. LRECs express ER-α and PR and incorporate ^3^H-TdR into their nuclei. Immunohistochemistry for ER-α and PR, as well as labeling for ^3^H-TdR, was performed on the intact mammary glands (thoracic) of female mice. Epithelial cells present in the glands expressed ER-α (**A-C**, red arrow) as well as PR (**D-F**, red arrow) in the NSP2 (A, D), NSP3 (B, E), and SP3 (C, F) groups. ^3^H-TdR was incorporated into the nucleus of some cells expressing the steroid receptors (green arrow, inset). Scale bars equal 20 μm. Click here for file

Additional file 2 **Supplemental figure S2**. Label-retaining cells are present in nonmammary tissues. Cells expressing 5-bromo-2-deoxyuridine were found in **(a) **cartilage, **(b) **adipose tissue, **(c) **skeletal muscle, and **(d) **periductal cells. Scale bars equal 20 μm. Click here for file
