# Supplementary figures and images for: Immortalized, premalignant epithelial cell populations contain long-lived, label-retaining cells that asymmetrically divide and retain their template DNA
Source: Breast Cancer Res. 2010 Oct 21;12(5):R86. doi: 10.1186/bcr2754 (PMC3096979; doi:10.1186/bcr2754)

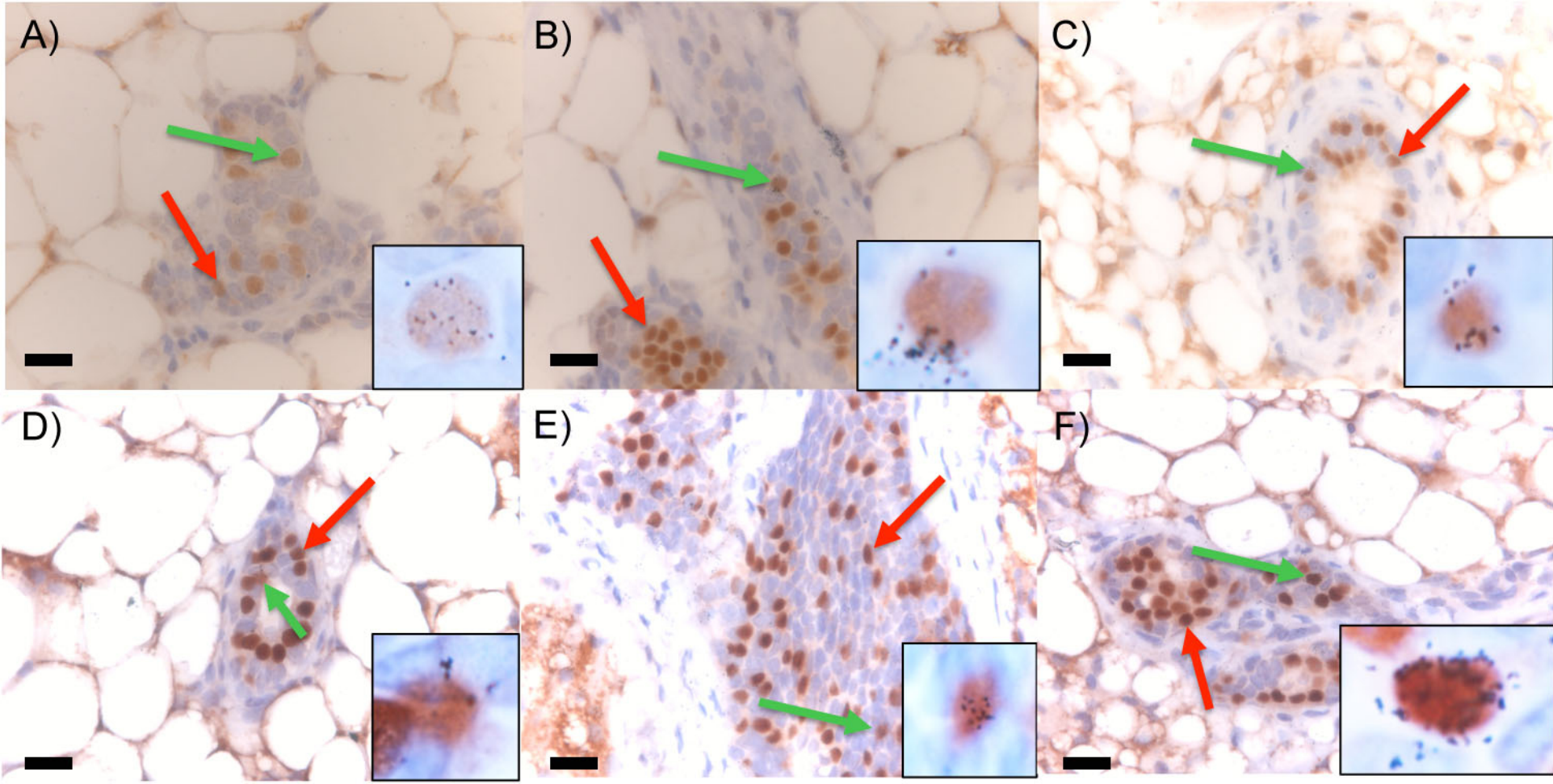

Supplement: Additional file 1 — Supplemental figure S1. LRECs express ER-α and PR and incorporate 3H-TdR into their nuclei. Immunohistochemistry for ER-α and PR, as well as labeling for 3H-TdR, was performed on the intact mammary glands (thoracic) of female mice. Epithelial cells present in the glands expressed ER-α (A-C, red arrow) as well as PR (D-F, red arrow) in the NSP2 (A, D), NSP3 (B, E), and SP3 (C, F) groups. 3H-TdR was incorporated into the nucleus of some cells expressing the steroid receptors (green arrow, inset). Scale bars equal 20 μm. [file bcr2754-S1.PDF]

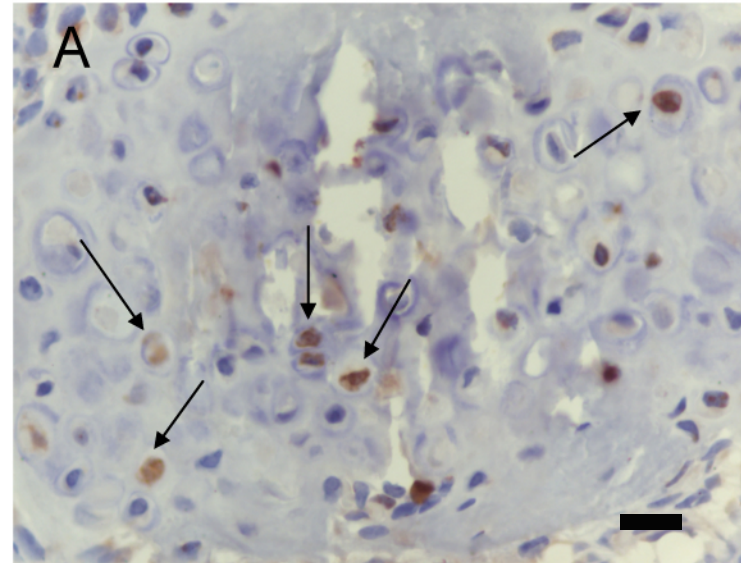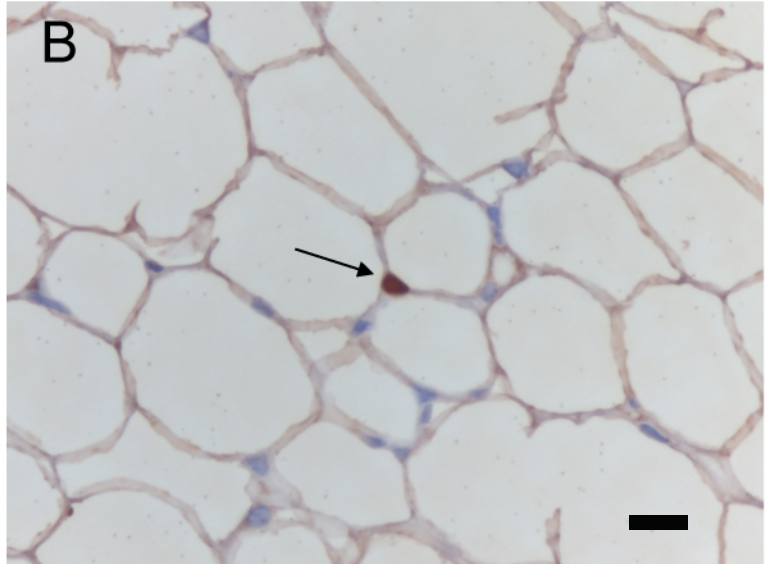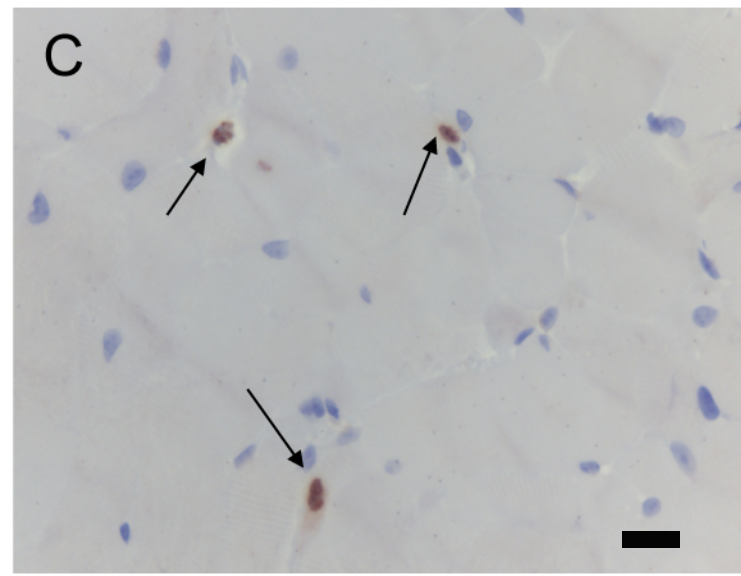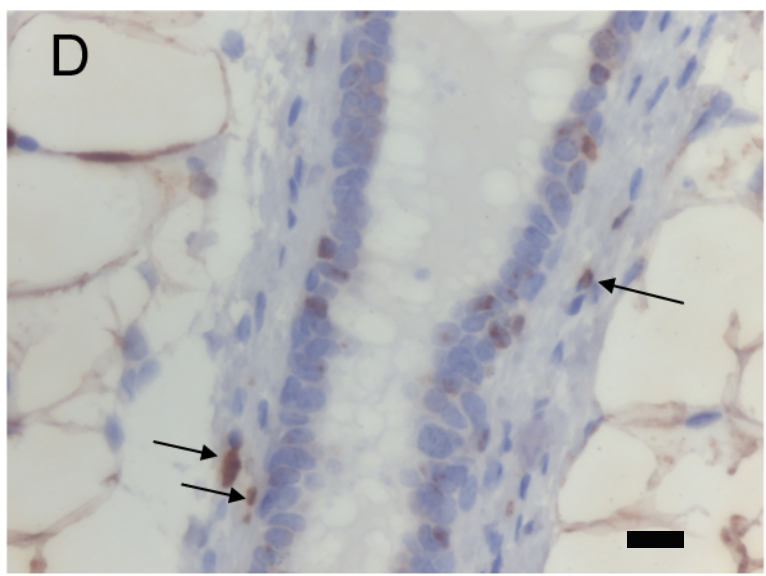

Supplement: Additional file 2 — Supplemental figure S2. Label-retaining cells are present in nonmammary tissues. Cells expressing 5-bromo-2-deoxyuridine were found in (a) cartilage, (b) adipose tissue, (c) skeletal muscle, and (d) periductal cells. Scale bars equal 20 μm. [file bcr2754-S2.PDF]
